# CAR-T manufacturing reduces heterogeneity between CIDP and multiple myeloma patient-derived T cells

**DOI:** 10.7150/thno.125983

**Published:** 2026-01-22

**Authors:** Xu Wang, Pu Wang, Qi Zhou, Xianzheng Wei, Yuhang Jin, Ying Liao, Xuan Zhao, Rui Hou, Sijin Li, Zhangchun Guan, Wen Ma, Dan Liu, Ming Shi

**Affiliations:** 1 Cancer Institute, Xuzhou Medical University, 209 Tongshan Road, Xuzhou, Jiangsu, 221004, China.; 2 Center of Clinical Oncology, The Affiliated Hospital of Xuzhou Medical University, 99 Huaihai Road, Xuzhou, Jiangsu, 221002, China.; 3 Jiangsu Center for the Collaboration and Innovation of Cancer Biotherapy, Xuzhou Medical University, 209 Tongshan Road, Xuzhou, Jiangsu, 221004, China.; 4 College of Pharmacy, Xuzhou Medical University, Xuzhou, Jiangsu, China.; 5 Department of Oncology, Central Hospital Affiliated to Shandong First Medical University, Jinan, Shandong, 250013, China.

**Keywords:** CAR-T cell therapy, T cell heterogeneity, autoimmune diseases, transcriptomic reprogramming, MM, CIDP

## Abstract

**Rationale:​​** CAR-T cell therapy has demonstrated remarkable promise for managing specific autoimmune disorders. However, it remains unclear, whether long-term immunosuppressive therapy in autoimmune patients adversely affects the phenotype and function of patient-derived CAR-T products. This study aimed to compare the characteristics of T cells and manufactured CAR-T cells from patients with multiple myeloma (MM) and chronic inflammatory demyelinating polyneuropathy (CIDP).

​​**Methods:​​** T cells isolated from MM and CIDP patients, as well as healthy volunteers (for baseline comparisons only), were analyzed. CAR-T cells were generated using an identical manufacturing process. A comprehensive analysis was conducted, including flow cytometry for phenotypic and functional assessment, transcriptomic profiling via RNA sequencing, and *in vitro* functional assays such as cytokine secretion and cytotoxicity tests.

​​**Results:​​** T cells from CIDP patients showed phenotypes and functional profiles more comparable to those from healthy volunteers. In contrast, MM-derived T cells showed increased CD8⁺ T cell frequency, elevated exhaustion markers, reduced naïve and less-differentiated subsets, and enhanced effector molecule production upon non-specific stimulation. CAR-T manufacturing reduced these inherent differences, yielding similar differentiation states, transcriptomic profiles, and convergent cytotoxic capacities. However, distinct immunomodulatory features persisted, as CIDP-derived CAR-T cells displayed reduced activation markers and lower IFN-γ secretion upon antigen stimulation compared to MM-derived CAR-T cells.

​​**Conclusions:​​** Our study reveals that CAR-T manufacturing process can reduce pre-existing T-cell heterogeneity across different patient populations. These findings support the feasibility of autologous CAR-T therapies in immunosuppressed autoimmune patients, demonstrating that critical cytolytic functions are preserved despite residual alterations in cytokine profiles.

## Introduction

Originally developed for oncology, CAR-T cell therapy is now being explored for autoimmune diseases (ADs), leveraging its consistent mechanism of targeting and eliminating pathogenic antigen-presenting cells 1, 2. In 2021, CD19-targeted CAR-T cells demonstrated exceptional efficacy in systemic lupus erythematosus (SLE), achieving sustained remission 3. Subsequent administration to five refractory SLE patients in 2022 induced progression-free survival exceeding two years, even after the cessation of immunosuppressive therapy, suggesting potential disease modification 4. By 2023-2024, clinical success extended to myositis, interstitial lung disease, neuromyelitis optica, and myasthenia gravis, with sustained therapeutic responses observed across these conditions 5-9. Taken together, these results underscore the transformative capacity of CAR-T therapy in revolutionizing care for refractory autoimmune disorders, offering the prospect of deep immune resetting and durable drug-free remission.

The cornerstone of autoimmune disease therapy involves the prolonged use of glucocorticoids 10-12 and immunosuppressive drugs, known to dampen TCR signaling and impede the activation and expansion of T cells 13-15. Current CAR-T therapy primarily targets relapsed and refractory autoimmune patients, a population characterized by extensive exposure to complex, prolonged drug regimens. Given the highly personalized nature of CAR-T therapy, their efficacy is intrinsically linked to autologous T-cell fitness 16-21. Critical knowledge gaps persist regarding whether T cells from such patients retain functional competence, and more fundamentally, whether CAR-T products manufactured from these cells show sustained phenotypic and functional integrity. While existing CAR-T manufacturing protocols-developed using cancer patient-derived T cells-provide optimal control benchmarks for evaluating autoimmune patient-derived counterparts, rigorous comparative analysis remains exceptionally challenging 22, 23. Beyond inherent T-cell source differences, variables including scFv sequence, CAR vector architecture, T-cell activation with CD3 and CD28 beads, expansion in IL-2-supplemented media, and lentiviral transduction must be strictly controlled.

Previously, our group engineered a bispecific CAR-T therapy directed at both BCMA and CD19 antigens (BC19 CAR-T). In a clinical study involving 50 subjects with relapsed and refractory multiple myeloma (r/r MM), we observed a 92% overall response rate. Furthermore, the median progression-free survival (PFS) and overall survival (OS) were both established at 19.7 months 24-26. This trial revealed transient B-cell depletion without severe adverse events following BC19 CAR-T infusion. Building on this safety and efficacy profile, we expanded clinical investigations to autoimmune diseases such as CIDP, myasthenia gravis (MG), and neuromyelitis optica, demonstrating encouraging therapeutic outcomes 27. We also reported a case involving CIDP, a demyelinating autoimmune condition. Following the infusion of BC19 CAR-T cells, the patient achieved treatment-free remission lasting over 3 years, allowing for the complete cessation of glucocorticoids and immunosuppressive drugs. This case represents the first globally documented report of clinically meaningful CAR-T efficacy in CIDP 28. Recently, multiple independent studies have reported the successful treatment of CIDP using BCMA-targeted CAR-T cells, with therapeutic mechanisms further supported by multi-omics analyses 29, 30. Notably, these studies include the use of the commercial CAR-T product, Ciltacabtagene autoleucel 31. Results from our team's ongoing trial involving multi-case CIDP analyses are forthcoming. This clinical experience provided a unique opportunity to benchmark the phenotypic and functional integrity of CAR-T cells manufactured from autoimmune patient-derived T cells against cancer patient-derived counterparts.

This study was designed to address whether prolonged immunosuppression in autoimmune patients impairs T-cell suitability for CAR-T therapy. We examined: (1) the ability of CIDP-derived T cells to yield functional CAR-T products akin to those from MM patients, and (2) the extent to which manufacturing reduces baseline T-cell differences. We hypothesized that the CAR-T manufacturing process would remodel the distinct baseline transcriptional and phenotypic landscapes, driving a functional convergence between CIDP- and MM-derived products.

## Materials and Methods

### ​​Ethical compliance and primary samples​​

The study protocol received formal clearance from the Medical Ethics Committee at the Affiliated Hospital of Xuzhou Medical University (XYFY2022-KL057-02; XYFY2020-KL062-01). All procedures adhered strictly to the ethical principles outlined in the Declaration of Helsinki. Peripheral blood mononuclear cells (PBMCs) derived from MM (*n* = 5) and CIDP (*n* = 5) cohorts, as well as healthy donors (HCs, *n* = 5) were isolated via leukocyte apheresis after obtaining written informed consent. Age data were available for 4 HC donors (29, 33, 45, and 55 years; median age 39), while demographic data for one donor was not retained. Clinical trials were registered at Chictr.org.cn (ChiCTR2200061267, ChiCTR2000033567). Healthy volunteer T cells were included solely for baseline phenotypic and functional assessments; ethical constraints precluded their use in CAR-T manufacturing. Detailed clinical characteristics, including the dosage and duration of glucocorticoid and immunosuppressive therapies for each patient, are provided in Table 1.

### ​​Cell lines and culture​​

U266 and NALM6 cells (Cell Bank of Chinese Academy of Sciences) were cultured in RPMI 1640 supplemented with 15% or 10% FBS, respectively. U266*^BCMA^*^-MUT-OE^ cells were generated to stabilize BCMA expression. All cells were STR-validated and confirmed mycoplasma-free.

### ​​GMP-grade CAR-T manufacturing​​

T cells isolated from PBMCs (EasySep™ Human T Cell Isolation Kit, STEMCELL cat#100-0695) were activated with CTS™ Dynabeads™ CD3/CD28 (ThermoFisher cat#40203D). After 48-72 h, cells were transduced with GMP-grade BC19 CAR lentivirus (Shanghai Jikai Biotechnology) at MOI = 5. For cell expansion, we utilized X-VIVO 15 medium supplemented with 200 U/mL IL-2, along with 5 ng/mL each of IL-7 and IL-15. To determine transduction efficiency, flow cytometry was performed between days 7 and 10. Detailed gating strategies for all flow cytometric analyses are presented in Figure S4-S8.

### ​​Flow cytometry analysis

#### Surface and intracellular staining

To assess cell viability, aliquots of 1 × 10⁶ cells were labeled using Zombie UV™ (BioLegend, cat#423107). Prior to surface staining, non-specific binding was inhibited by incubating cells with Human TruStain FcX™ (BioLegend, cat#422301). Subsequently, surface antigens were labeled by incubating cells with fluorochrome-conjugated antibodies for 30 min at 4 °C. Regarding intracellular cytokine analysis, cellular activation was achieved via stimulation with 100 ng/mL PMA and 1 μM ionomycin for 2 h, followed by the addition of GolgiPlug™ (BD Biosciences, cat#555029) for an additional 4 h. Finally, cells were processed using a fixation/permeabilization kit (BioLegend, cat#420801) before intracellular staining.

Antibody panels:* T cell subsets​​:* CD45-PE (BioLegend, clone HI30, cat#304008), CD3-APC (BioLegend, clone HIT3a, cat#300312), CD4-FITC (BioLegend, clone OKT4 or SK3, cat#317408 or 344604), CD8-APC-Cy7 (BioLegend, clone HIT8a, cat#300926). *Memory phenotype***​**​: CD45RO-BV785 (BioLegend, clone UCHL1, cat#304234), CCR7-PE (BioLegend, clone G043H7, cat#353203). *Exhaustion markers***​**​: PD1-PE-Cy7 (BioLegend, clone A17188B, cat#621615), TIM-3-APC (BioLegend, clone F38-212, cat#345011). *Cytokine secretion​​*: IFN-γ-PE (BioLegend, clone 4S.B3, cat#502510), TNF-α-PE-Cy7 (BioLegend, clone MAb11, cat#502929), IL-2-APC (BioLegend, clone MQ1-17H12, cat#500309). *Activation:* CD25-APC (BioLegend, clone BC96, cat#302609), CD69-PE-Cy7 (BioLegend, clone FN50, cat#310911) assessed at 24 h. *CAR detection​​:* Protein L (ACROBiosystems, cat#RPL-P814R) + Brilliant Violet 421™ Streptavidin (BioLegend, cat#405226). Data were acquired on a BD LSRFortessa and analyzed using FlowJo v10.9.0.

#### Co-culture experiments in single and multiple round cultures

For target cell discrimination, CellTracker™ Deep Red Dye (1:8000, ThermoFisher, cat#C34565) was used to label both U266*^BCMA^*^-MUT-OE^ and NALM-6 cells. Residual targets quantified using CountBright™ Absolute Counting Beads (ThermoFisher, cat#C36950). In single-round co-culture experiments, 2 × 10^5^ CIDP-CAR-T and MM-CAR-T were co-cultured with U266*^BCMA^*^-MUT-OE^ cells and NALM-6 cells at E : T = 1:5 and 1:10, and flow cytometry was performed after 24 h of co-culture. In multiple rounds co-culture experiments, CAR-T cells were challenged with unlabeled U266*^BCMA-MUT-OE^* and NALM-6 in rounds 1 and 2, followed by fluorescently labeled tumor cells in round 3. Specific lysis of the labeled targets was assessed 24 h later to determine residual killing capacity. CAR^+^ T cells were incubated with anti-human CD3 conjugated to PerCP-Cy5.5 (1:100, Biolegend, clone OKT3, cat#317335), Protein L (1:400, ACROBiosystems, cat#RPL-P814R), and Brilliant Violet 421™ Streptavidin (1:1000, Biolegend, cat#405226), and tumor cells were detected using the Deep Red channel. For the quantification of surviving tumor cells, samples were spiked with 1 μL of CountBright™ Absolute Counting Beads (ThermoFisher, cat#C36950).

### Degranulation and perforin detection​​

1 × 10⁶ CAR-T cells (CIDP or MM-derived) were co-cultured with target cells at E : T = 1:1. After 2 h, GolgiPlug™ (BD Bioscience, cat# 555029) and anti-CD107a-PE (BioLegend, clone H4A3, cat# 328607) were added. Cells were harvested after 4 h total co-culture, stained with Zombie UV™ (BioLegend, cat# 423107), followed by surface CAR detection (Protein L, cat#RPL-P814R + BV421-Streptavidin, cat# 405226). After fixation/permeabilization, intracellular perforin was stained with PE-Cy7-conjugated antibody (BioLegend, clone B-D48, cat# 353315).

### Bulk RNA-seq sequencing

Total RNA isolated from 5 × 10⁶ T or CAR-T cells underwent library preparation using oligo(dT) magnetic beads (Thermo Fisher, cat#25-61005) for mRNA enrichment. Libraries were constructed via magnesium fragmentation (NEB, cat#E6150S), cDNA synthesis, and end repair, yielding 300 ± 50 bp fragments. Paired-end 150 bp sequencing was conducted using the Illumina Novaseq™ 6000 platform (LC Bio). Raw reads were processed to FPKM values, with differential expression analyzed in R (v4.3.2) using DESeq2 (|Log₂Fc| ≥ 1.5, adj. *p < 0.05*).

### TCSS score calculation

We employed the TCellSI algorithm to characterize the functional landscape of T cells and CAR-T cells. Relying on specific gene sets, this tool evaluates eight functional categories: Senescence, Terminal exhaustion, Progenitor exhaustion, Cytotoxicity, Helper, Proliferation, Regulating, and Quiescence 32. Bulk RNA-seq transcriptomes from all samples were analyzed using the TCellSI package (v2.0.1) with default parameters. Enrichment scores for each state were calculated as the mean expression of curated signature genes (minimum 15 genes per state), z-score normalized across the cohort. We applied a significance threshold of adjusted *p < 0.05* (corrected via the Benjamini-Hochberg method) to identify enriched states.

### Statistical analysis

All data were analyzed using GraphPad Prism 9.4.0 software. Given the exploratory nature of this study and the rarity of the CIDP clinical specimens, the sample size was determined based on availability and feasibility for deep-phenotyping assays rather than an a priori power calculation. Consequently, non-parametric tests were primarily used to ensure statistical robustness despite the limited cohort size. For time-course data **(Figure 4B)**, a Two-way ANOVA with repeated measures (or Mixed-effects model) was used. The statistical results were presented as mean ± standard deviation (SD). A *P-value* of less than 0.05 was considered statistically significant. Comparisons yielding non-significant *p-values* indicate a failure to detect a difference under these conditions, rather than statistical proof of equivalence.

## Results

### CIDP derived T-cell phenotypes mirror healthy controls

**Figure 1:**
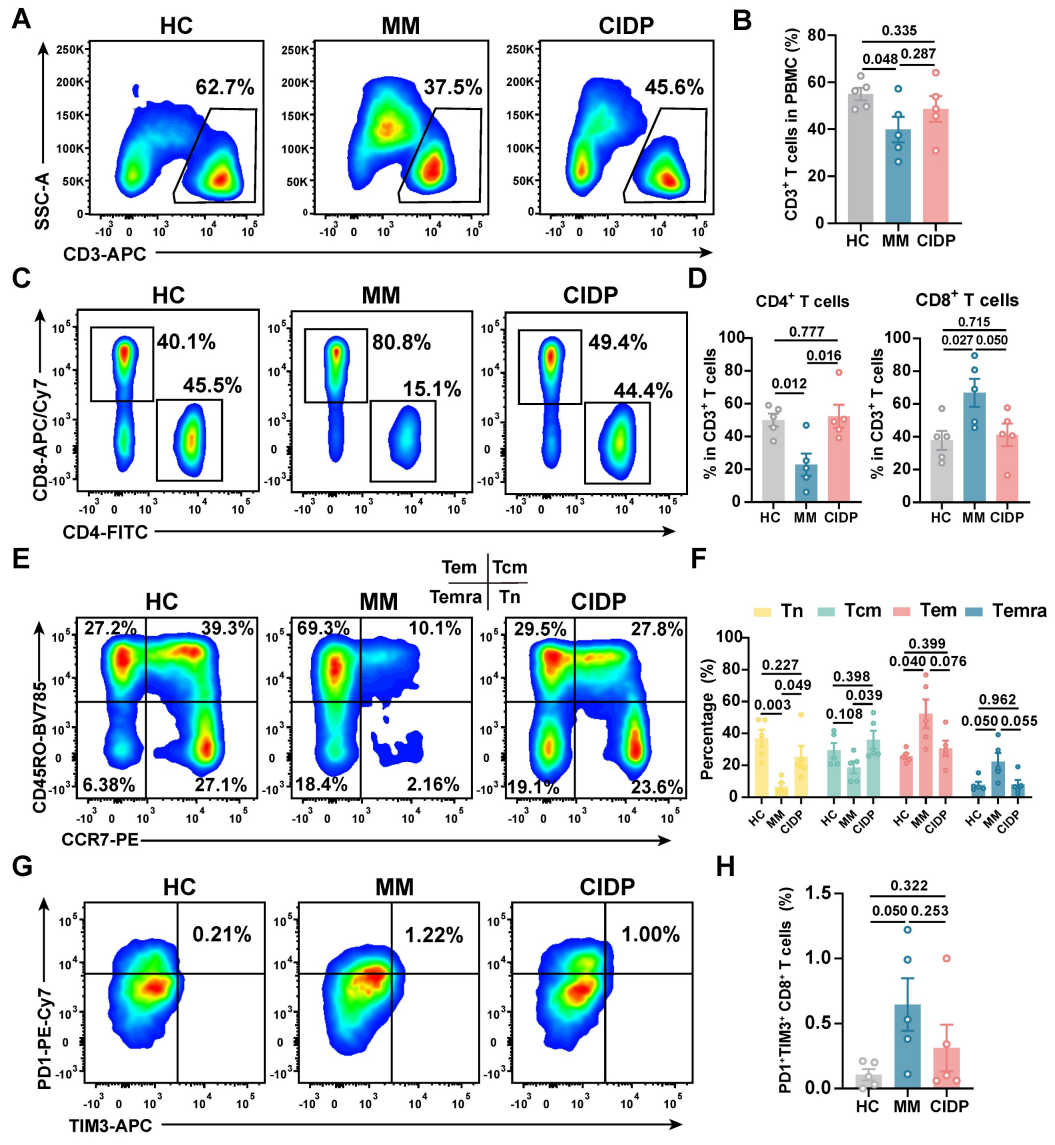
** CIDP patient-derived T cells demonstrate phenotypes more akin to healthy controls than MM patient-derived T cells.​​** Healthy donor-derived PBMCs (HCs,* n* = 5), multiple myeloma (MM, *n* = 5), and chronic inflammatory demyelinating polyneuropathy (CIDP, *n* = 5) patients were analyzed by flow cytometry. (A) Representative flow cytometry plots and (B) quantification of CD3^+^ T cell frequency in PBMCs. (C) Representative plots and (D) quantification of CD4^+^ and CD8^+^ T cell subsets within CD3^+^ T cells. (E) Representative plots and (F) quantification of naive (Tn, CCR7^+^CD45RO^-^), central memory (Tcm, CCR7^+^CD45RO^+^), effector memory (Tem, CCR7^-^CD45RO^+^), and terminally differentiated effector memory (Temra, CCR7^-^CD45RO^-^) T cells. (G) Representative plots and (H) quantification of PD-1^+^Tim-3^+^ exhausted CD8^+^ T cells. Data are presented as mean ± SD. A *P-value* of less than 0.05 was considered statistically significant.

To enable rigorous comparison of phenotypic and functional differences in patient-derived T-cell subsets, we included five healthy volunteers (HCs) as baseline controls. Clinically, all enrolled MM and CIDP patients (*n* = 5 per cohort) achieved complete remission after receiving the CAR-T products manufactured from their T cells. Comprehensive clinical profiles, including prior treatment histories, are detailed in **Table 1**. CAR-T products were manufactured from autologous T cells, where the CD3⁺ T-cell frequency and CD4⁺:CD8⁺ ratio in patient PBMCs critically impact manufacturing success and CAR-T potency and persistence 17-19, 33. Relative to HCs, MM patients exhibited significantly reduced CD3⁺ T-cell frequencies. CD3⁺ frequencies in CIDP patients were numerically lower, although not statistically significant **(Figure 1A-B)**. Notably, CIDP patients maintained CD4⁺ and CD8⁺ T-cell proportions comparable to HCs despite chronic immunosuppression. In contrast, MM patients showed significantly decreased CD4⁺ T-cell percentages and increased CD8⁺ T-cell percentages compared to both HCs and CIDP patients **(Figure 1C-D)**.

Given the established link between less-differentiated memory phenotypes and enhanced therapeutic efficacy and persistence, alongside the detrimental impact of T-cell exhaustion, we performed detailed comparisons of memory and exhaustion markers. Memory T-cell compartments in CIDP patients did not significantly differ from HCs. In contrast, the MM cohort was characterized by a marked decrease in the proportion of naïve T cells, concurrent with an expansion of terminally differentiated effector memory T cells (Temra) **(Figure 1E-F)**. Furthermore, MM patients showed a trend toward higher proportions of PD1⁺Tim3⁺ exhausted and terminally differentiated T cells compared to HCs, even though it did not meet the threshold for significance **(Figure 1G-H)**. Overall, our findings indicate that T-cell phenotypes in CIDP patients more closely resemble those in HCs while differ from MM patients.

### CIDP-derived T cells display healthy-like maximal effector potential, contrasting with the hyper-functional phenotype of MM

**Figure 2:**
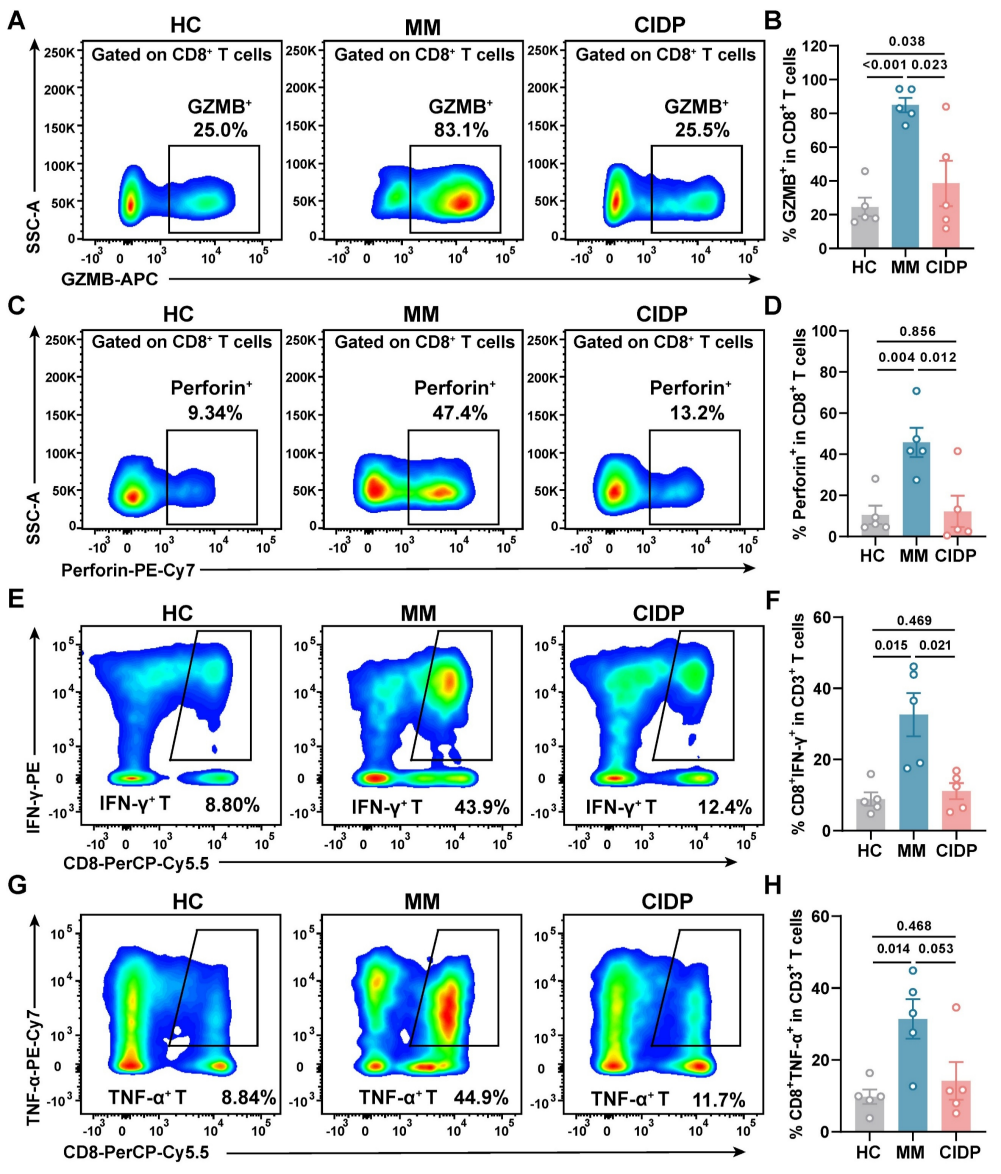
** CIDP-derived T cells display healthy-like maximal effector potential, contrasting with the hyper-functional phenotype of MM.​​** T cells from HCs (*n* = 5), MM (*n* = 5), and CIDP (*n* = 5) patients were stimulated with 100 ng/mL PMA and 1 μM ionomycin for 6 h. Cytotoxic function and cytokine production were assessed. Representative flow cytometry plots of (A) Granzyme B^ +^ and (C) Perforin^+^ T in CD8^+^ T cells. Quantification of (B) Granzyme B^ +^ and (D) Perforin^+^ T in CD8^+^ T cells. Representative flow cytometry plots of (E) IFN-γ^+^ and (G) TNF-α^+^ T in CD8^+^ T cells. Quantification of (F) IFN-γ^+^ and (H) TNF-α^+^ in CD8^+^ T cells. Data are presented as mean ± SD. A *P-value* of less than 0.05 was considered statistically significant.

To assess the maximum potential for cytokine production of T cells derived from various sources, we activated the cells using PMA and ionomycin for a period of 6 h. Analysis revealed that T cells from CIDP patients released Granzyme B **(Figure 2A-B)** and perforin **(Figure 2C-D)** at levels comparable to HCs​​, while MM-derived T cells showed significantly elevated production compared to both groups. ​​Similarly, the proportion of IFN-γ⁺ **(Figure 2E-F)** and TNF-α⁺ **(Figure 2G-H)** CD8⁺ T cells in CIDP patients showed trends similar to HCs​​, contrasting with the significantly increased frequencies observed in MM patients. CIDP-derived T cells maintained maximal effector molecule production capacities comparable to HCs. This profile stands in marked contrast to the phenotype observed in MM-derived T cells, characterized as terminally differentiated with high effector potential, evidenced by elevated maximal production of cytotoxic granules and cytokines. This assay measures the maximal capacity of T cells to produce effector molecules, acting as a measure of cellular competence rather than physiological antigen-specific cytotoxicity.

### ​​Distinct T-Cell transcriptional landscapes in MM versus CIDP highlight proximity of CIDP to healthy T cells

**Figure 3:**
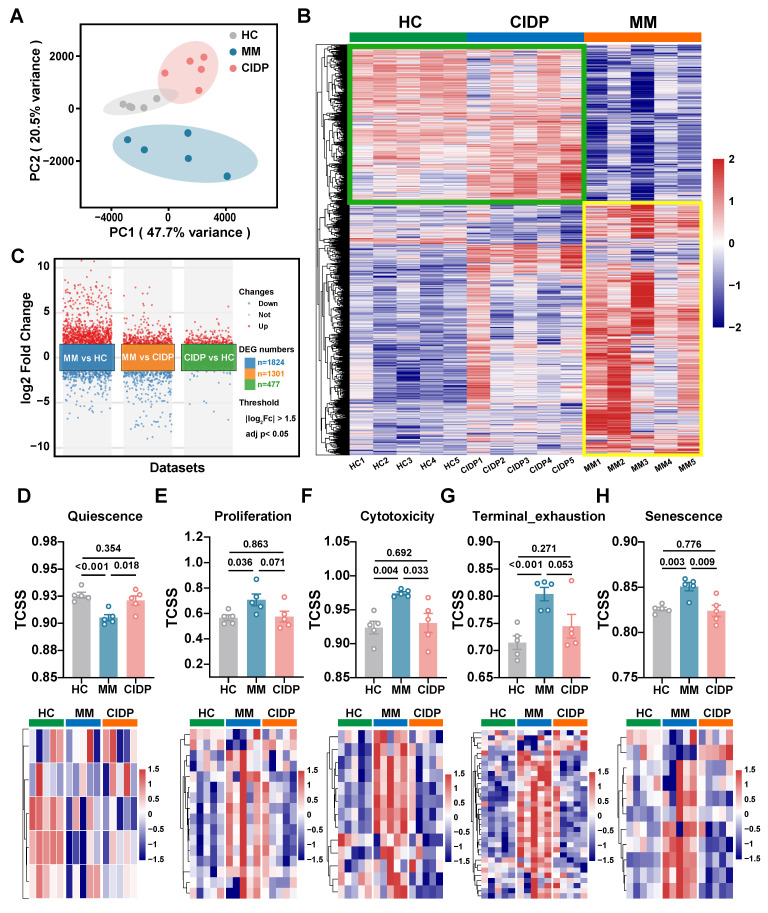
** Transcriptomic profiling reveals T cells from CIDP patients align closely with healthy controls, while MM-derived T cells are distinct.​​ Bulk RNA sequencing was performed on T cells isolated from HCs (*n* = 5), MM (*n* = 5), and CIDP (*n* = 5) patients. (A) Principal component analysis (PCA) plot of transcriptomes.** (B) Heatmap of hierarchical clustering based on differentially expressed genes (DEGs; |log_2_Fc| ≥ 1.5, adj. *p < 0.05*) across all groups. (C) Volcano plot of DEGs in MM vs HC, MM vs CIDP and CIDP vs HC groups. Scatter plot and heatmaps depicting expression levels of signature genes for selected T cell states: (D) Quiescence, (E) Proliferation, (F) Cytotoxicity, (G) Terminal exhaustion and (H) Senescence. Data in (D-H) are presented as mean ± SD. Data are presented as mean ± SD. A *P-value* of less than 0.05 was considered statistically significant.

To provide a transcriptomic basis for the distinct phenotypic and functional profiles observed, we conducted RNA sequencing on T cells obtained from the HC, MM, and CIDP cohorts. Principal component analysis (PCA) showed that while HC and CIDP samples formed distinct clusters, they grouped closer to each other than to the divergent MM samples **(Figure 3A)**. Hierarchical clustering of differentially expressed genes (DEGs) demonstrated a similar expression profile between HC and CIDP, contrasting sharply with the distinct profile of MM samples **(Figure 3B)**. Comparison of CIDP with HC identified 477 DEGs (404 upregulated, 73 downregulated). MM versus HC demonstrated significantly more DEGs, reaching 1,824 (1,208 upregulated, 616 downregulated) and exceeding 1301 (724 upregulated, 577 downregulated) found in CIDP **(Figure 3C)**. We employed the TCellSI algorithm to characterize transcriptomic variations, evaluating eight functional categories: Helper, Cytotoxicity, Proliferation, Regulating, Quiescence, Senescence, Progenitor exhaustion, and Terminal Exhaustion 32. Notably, the eight-state scores for CIDP-derived T cells showed no significant differences compared to HC. In contrast, MM-derived T cells showed a significantly reduced Quiescence score relative to HC, along with significantly elevated scores for Proliferation, Cytotoxicity, Terminal Exhaustion, and Senescence **(Figure 3D, Figure S1)**. This concurrent elevation of cytotoxicity and exhaustion signatures is consistent with the Temra-enriched phenotype observed in **Figure 1**, reflecting cells that are terminally differentiated yet retain high effector gene expression. Heatmaps depicting signature genes for each state further highlighted the close similarity between CIDP and HC T cells, while MM samples showed significant divergence from both HC and CIDP groups **(Figure 3E-H, Figure S1)**. These transcriptomic analyses confirm that CIDP-derived T cells parallel HCs at both transcriptomic and cellular levels, while MM-derived T cells demonstrated distinct differences.

### ​​CAR-T manufacturing reduces T-cell phenotype heterogeneity

**Figure 4:**
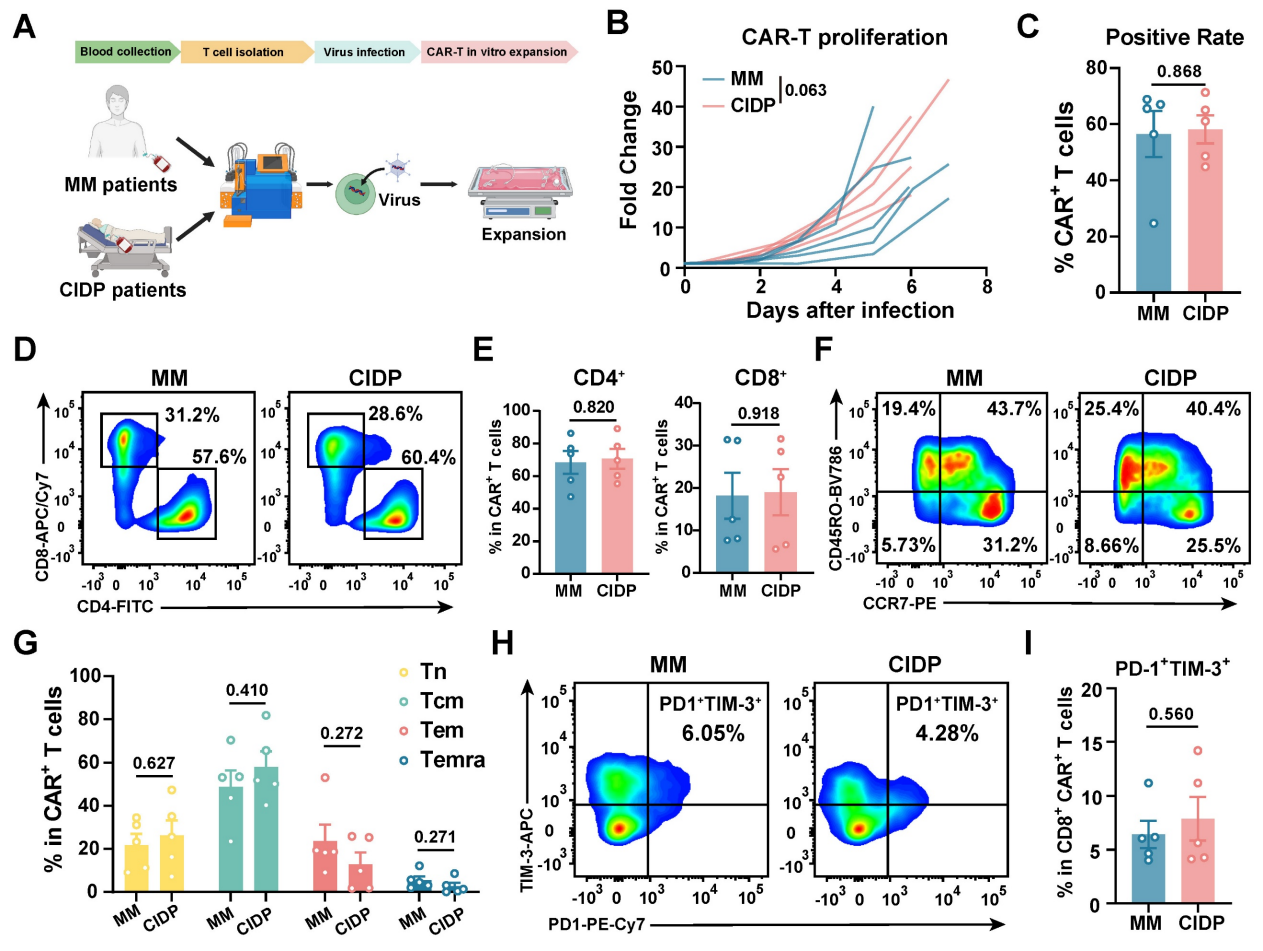
** CAR-T cell manufacturing reduces phenotypic heterogeneity observed in source T cells.​​** CAR-T cells were manufactured from MM (MM-CAR-T, *n* = 5) and CIDP (CIDP-CAR-T, *n* = 5) patient T cells using identical GMP processes. (A) Schematic of CAR-T manufacturing and phenotyping. (B) *In vitro* proliferation kinetics of MM-CAR-T and CIDP-CAR-T cells over 7 days. (C) CAR positivity rate determined by Protein L staining on day 7. (D) Representative flow cytometry plots and (E) quantification of CD4^+^ and CD8^+^ subsets within CAR^+^ T cells. (F) Representative plots and (G) quantification of naive (Tn), central memory (Tcm), effector memory (Tem), and effector (Temra) phenotypes within CAR^+^ T cells. (H) Representative plots and (I) quantification of PD-1^+^Tim-3^+^ exhausted CD8^+^CAR^+^ T cells. Data are presented as mean ± SD. Statistical significance for proliferation kinetics (B) was determined by Two-way ANOVA with repeated measures. A *P-value* of less than 0.05 was considered statistically significant.

To evaluate whether chronic immunosuppression compromises CAR-T cell quality, we generated CAR-T cells from MM and CIDP patients using an identical protocol 24, and named them MM-CAR-T and CIDP-CAR-T, respectively** (Figure 4A)**. However, ethical approval for using T cells from healthy volunteers to manufacture CAR-T cells was not obtained. We monitored the *in vitro* proliferation and positivity rate of the two types of CAR-T cells, which showed similar proliferation trends, although MM-CAR-T cells showed a tendency for higher expansion **(*p* = 0.063; Figure 4B)** and an average positivity rate above 50% **(Figure 4C)**. Unlike the previously observed decrease in CD4^+^ T cells and increase in CD8^+^ T cells in T cells from MM patients, the proportions of CD4^+^ and CD8^+^ T cells in the two CAR-T products were comparable, with no statistically significant differences detected **(Figure 4D-E)**. In addition to these observations, we also focused on the memory and exhaustion phenotypes of CAR-T products. Central memory T cells and less differentiated subpopulations are more favorable for CAR-T cell expansion, survival, and long-term persistence *in vivo* 34-36. The higher the proportion of stem-like or central memory cells in the CAR-T product, the better the therapeutic persistence 37. Similar to the consistent ratios of CD4 and CD8 T cells, the increased differentiation and exhaustion of T cells previously observed in MM patients were not recapitulated in CAR-T products. Conversely, the memory and exhaustion profiles of both CAR-T preparations were indistinguishable, yielding no statistically significant variations in the frequency of Tn, Tcm, Tem, and Temra subpopulations **(Figure 4F-I)**. These findings indicate that the manufacturing process markedly reduced T-cell heterogeneity, driving phenotypic convergence between the two cohorts.

### CIDP-CAR-T demonstrate reduced activation but comparable cytotoxicity

**Figure 5:**
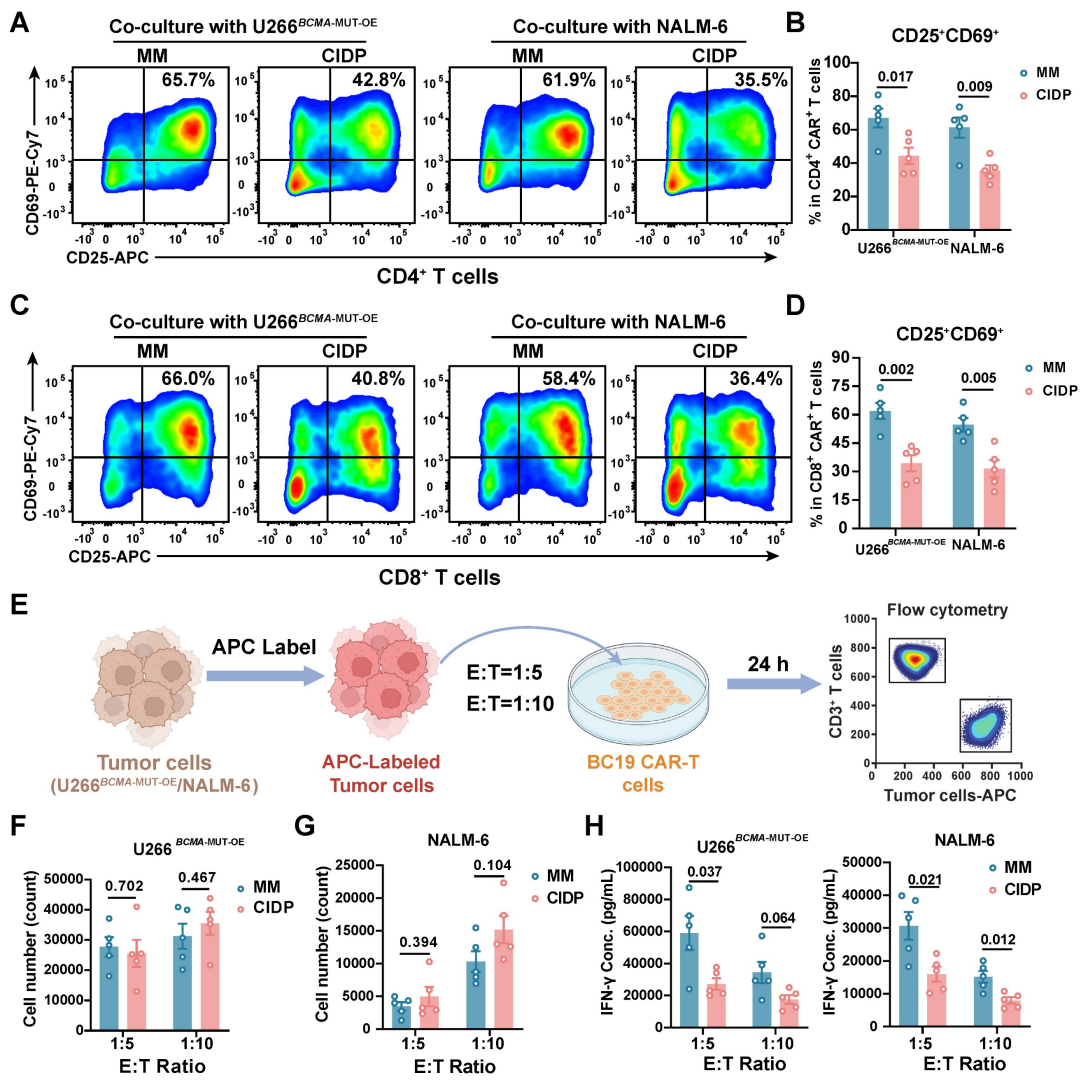
** CIDP-CAR-T cells show reduced activation and IFN-γ secretion but comparable cytotoxicity to MM-CAR-T cells upon antigen encounter.​​** MM-CAR-T (*n* = 5) and CIDP-CAR-T (*n* = 5) cells were incubated in the presence of target cells expressing BCMA (U266*^BCMA^*^-MUT-OE^) or CD19 (NALM-6). Representative flow plots and quantification of CD25^+^CD69^+^ activated (A-B) CD4^+^CAR^+^ and (C-D) CD8^+^CAR^+^ T cells following a 24-hour co-incubation period. (E) Schematic of single-round cytotoxicity assay. Cytotoxicity against (F) U266*^BCMA^*^-MUT-OE^ and (G) NALM-6 target cells at effector-to-target (E : T) ratios of 1:5 and 1:10 after 24 h. Residual target cells were quantified using counting beads. Secreted (H) IFN-γ levels in co-culture supernatants of U266*^BCMA^*^-MUT-OE^ and NALM-6. Data are presented as mean ± SD. A *P-value* of less than 0.05 was considered statistically significant.

To further assess functional differences between the two CAR-T cell types, we co-cultured them with BCMA- or CD19-positive target cells and measured activation levels. Consistent with initial concerns, CIDP-derived CAR-T cells showed a lower proportion of CD25^+^CD69^+^ T cells. This difference was observed in both CD4^+^ and CD8^+^ T cell subsets, indicating a persistent divergence in activation capacity between CIDP-CAR-T and MM-CAR-T cells following target antigen stimulation **(Figure 5A-D)**. This raised the question of whether, despite similar phenotypes, CIDP-CAR-T cells possess inferior cytotoxic function. To test this, we conducted co-culture assays with BCMA- or CD19-positive target cells across various effector-to-target (E : T) ratios to assess cytotoxicity **(Figure 5E)**. In single-round co-cultures, both CAR-T cells mediated killing of target cells with no statistically significant disparity observed between BCMA-positive and CD19-positive groups **(Figure 5F-G)**. At a low E : T ratio of 1:10, killing capacity against BCMA-positive U266 targets remained comparable **(Figure 5F)**. Against CD19-positive NALM-6 targets, a trend toward reduced killing was observed in CIDP-CAR-T cells (*p* = 0.104), though this did not reach statistical significance **(Figure 5G)**. However, importantly, this potential subtle divergence in single-round efficiency did not compromise long-term function, as evidenced by the preserved cumulative cytotoxicity in the sequential rechallenge assay** (Figure S2D)**. We further quantified cytokine secretion in co-culture supernatants. CIDP-CAR-T cells produced markedly reduced levels of IFN-γ relative to the MM-CAR-T group **(Figure 5H)**, whereas TNF-α and IL-2 levels remained comparable **(Figure S2A-B)**. Previous studies indicate that glucocorticoids selectively suppress the *IFNG* locus through transcriptional interference, such as the inhibition of T-bet, and epigenetic remodeling, without affecting *TNF* or *IL2* 10. Additionally, IFN-γ production is highly dependent on JAK-STAT1/STAT4 signaling, which may be inhibited by immunosuppressive drugs 11. Given that CIDP patients receive chronic glucocorticoid and immunosuppressive therapies, this may explain the attenuated IFN-γ release in CIDP-CAR-T cells. The preserved TNF-α and IL-2 secretion likely supports effector function, while the reduced IFN-γ represents a distinct functional alteration 38. Thus, while the manufacturing process did not fully erase, it successfully aligned the critical cytotoxic capabilities of CIDP-CAR-T cells with those of MM-CAR-T cells.

Our next objective was to assess whether the attenuated activation of CIDP-CAR-T cells and IFN-γ impact cytotoxic persistence under persistent antigen exposure. Sequential rechallenge assays were designed to model sustained antigen stimulation and potential cytotoxicity decline **(Figure S2C)**. Consistent with single-round assays, both CAR-T cell types maintained sustained cytotoxic activity, with no significant divergence observed between the groups across multiple rounds of stimulation **(Figure S2D)**. To further confirm functional parity, degranulation **(Figure S2E-F)** and perforin **(Figure S2G-H)** release were measured in co-cultured cells, yielding results concordant with the cytotoxicity assays. In summary, these results demonstrate that despite lower activation levels and reduced IFN-γ production in CIDP-CAR-T cells compared to MM-CAR-T counterparts, they display comparable *in vitro* cytotoxic potency, persistence, and degranulation capacities.

### CAR-T manufacturing drives transcriptional convergence in CIDP and MM T cells

**Figure 6:**
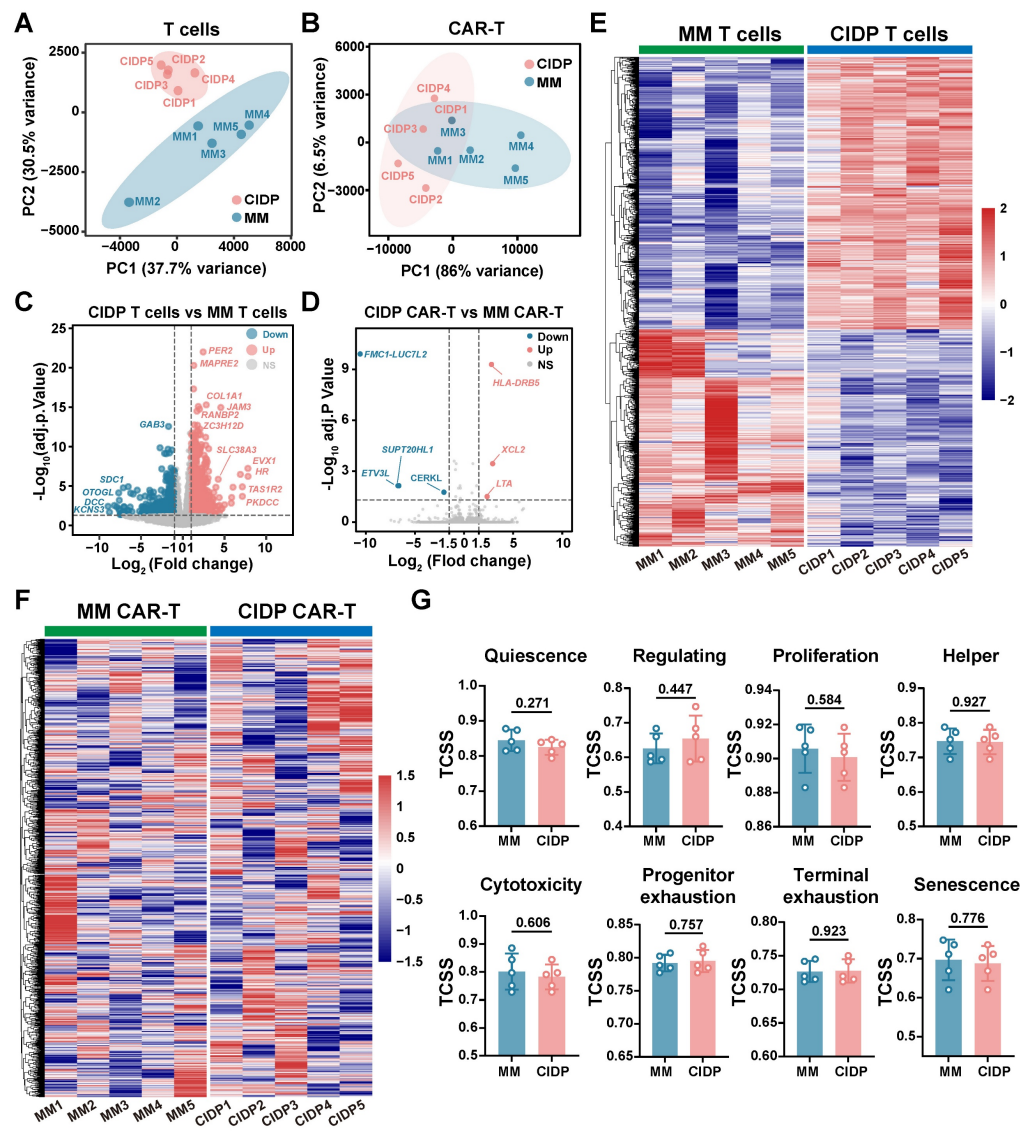
** CAR-T cell manufacturing drives transcriptional convergence of MM-derived and CIDP-derived T cells.​​** Bulk RNA sequencing was performed on source T cells and corresponding manufactured CAR-T cells from MM (*n* = 5) and CIDP (*n* = 5) patients. (A) PCA plot of transcriptomes from source MM T cells and CIDP T cells. (B) PCA plot of transcriptomes from MM-CAR-T and CIDP-CAR-T cells. Volcano plots depicting DEGs; |log_2_FC| ≥ 1.5, adj. *p < 0.05*) for (C) CIDP T cells vs MM T cells and (D) CIDP-CAR-T vs MM-CAR-T. (E) Heatmap of the 1,301 DEGs identified between source CIDP T cells and MM T cells. (F) Heatmap showing expression patterns of the 1,301 DEGs in CIDP-CAR-T and MM-CAR-T cells. (G) TCellSI algorithm scores for the eight functional T cell states in CAR-T cells. Data in (G) are presented as mean ± SD. A *P-value* of less than 0.05 was considered statistically significant.

To further elucidate the convergence in phenotype and function between CIDP-CAR-T and MM-CAR-T cells, transcriptomic profiling was conducted via RNA-seq on both CAR-T populations. We also compared transcriptomic profiles of T cells derived from both disease sources to directly visualize changes occurring during CAR-T manufacturing. Principal Component Analysis (PCA) revealed significant separation between T cells from CIDP and MM patients, with low dispersion among CIDP samples and high dispersion in the MM group **(Figure 6A)**. Conversely, PCA analysis of CAR-T cells revealed that the two groups remained transcriptionally distinct. Specifically, the MM-CAR-T group retained greater variance compared to the tightly clustered CIDP-CAR-T group. However, relative to the profound separation observed in source T cells, the inter-group distance was reduced post-manufacturing **(Figure 6B)**. Volcanic plot analysis identified numerous DEGs identified when comparing T cells isolated from CIDP and MM patients **(Figure 6C)**, but a marked reduction in the number of statistically identified DEGs between corresponding CIDP-CAR-T and MM-CAR-T cells **(Figure 6D)**. Given the limited sample size (*n* = 5), certain genes with notable fold-changes did not reach statistical significance, suggesting that some residual heterogeneity persists despite the overall trend toward convergence. The number of DEGs between patient-derived T cells reached 1,301, and heatmap visualization of these genes confirmed distinct transcriptional signatures distinguishing CIDP and MM T cells **(Figure 6E)**. Analysis of these 1,301 DEGs in CAR-T samples revealed a disorganized expression pattern with no clear clustering **(Figure 6F)**, indicating that the transcriptional program driving the initial disease-associated segregation was largely remodeled. Similarly, the evaluation of eight T cell states using the TCellSI algorithm demonstrated that CAR-T manufacturing markedly reduced the phenotypic and functional disparities identified within T cells. The prominent disparities initially observed in T cell state scores were markedly reduced, rendering the two CAR-T cohorts statistically indistinguishable **(Figure 6G, Figure S3)**. Collectively, these results transcriptionally confirm the robust convergence of phenotypes and cytotoxic functions in CIDP- and MM-derived T cells following their conversion into CAR-T cells.

## Discussion

A central finding of this study is that the CAR-T manufacturing process drives a remarkable phenotypic and transcriptomic alignment between MM- and CIDP-derived T cells. The observed convergence highlights the reprogramming capacity of CAR manufacturing. Activation and *ex vivo* expansion are known to remodel epigenetic landscapes 33, 39, 40. Our transcriptomic data extend this paradigm: the 1,301 DEGs distinguishing source T cells were largely aligned in CAR-T products, and divergent TCellSI scores were reduced **(Figure 6G)**. The robustness of this reprogramming was particularly evident given the cohort's baseline heterogeneity. Tracking individual PCA trajectories **(Figure 6A-B)** revealed that the manufacturing process effectively aligned diverse profiles, including that of patient MM-001 who received prior CAR-T therapy and patient MM-002 who showed baseline variance. This ability to minimize inter-patient distance despite varied clinical histories underscores the capacity of the manufacturing protocol to substantially remodel patient-specific heterogeneity.

Baseline comparisons revealed that CIDP-derived T cells resembled healthy donors, whereas MM T cells displayed a "paradoxical" phenotype of elevated exhaustion markers (PD-1^+^Tim-3^+^) alongside hyper-functional cytotoxic granule production **(Figure 2)**. This profile aligns with the characteristics of Terminally Differentiated Effector Memory (Temra) T cells, which arrest in the G1 phase but constitutively maintain high levels of effector molecules, a state often referred to as the senescence-associated secretory phenotype (SASP) 41-43. Therefore, the hyper-functionality observed in MM T cells reflects the high intracellular reserves of these terminally differentiated cells rather than a healthy plastic state. CAR manufacturing successfully mitigated these disparities, counteracting both immunosuppression- and malignancy-driven dysfunction.

Despite global convergence, a specific functional divergence persisted: CIDP-CAR-T cells showed reduced CD25 and CD69 expression and IFN-γ secretion compared to MM-CAR-T cells. This likely reflects residual epigenetic marks at specific loci, such as the *IFNG* gene, that were not fully erased, possibly due to chronic glucocorticoid exposure 10, rather than a global failure of reprogramming. Importantly, this immunomodulatory difference did not compromise direct cytotoxicity, which remained preserved across single-round and serial-killing assays. This uncoupling of potent killing from high cytokine release aligns with emerging strategies to mitigate cytokine release syndrome (CRS) 38, 44, 45, suggesting a potentially favorable safety profile for autoimmune applications.

​​Our study still has limitations. The small cohort size (*n* = 5 per group) constrains statistical power; thus, the absence of significant differences should be interpreted as a lack of detected divergence rather than proof of strict equivalence. Moreover, ethical constraints prevented the manufacturing of CAR-T cells from healthy volunteers. Without this key control, we cannot definitively determine whether the process "normalizes" cells to a healthy state or merely reduces inter-patient heterogeneity. Additionally, we acknowledge the age disparity between the younger healthy controls and the older MM cohort. As immunosenescence influences T-cell differentiation, this mismatch represents a potential confounder in our baseline phenotypic comparisons. Furthermore, while we did not perform *in vivo* xenograft comparisons, the clinical remission achieved in all enrolled CIDP patients serves as definitive autologous validation of functional potency, mitigating the lack of animal models. Future studies should address these gaps and further elucidate the mechanisms by which manufacturing overrides disease-specific epigenetic signatures.

## Conclusions

**Figure 7:**
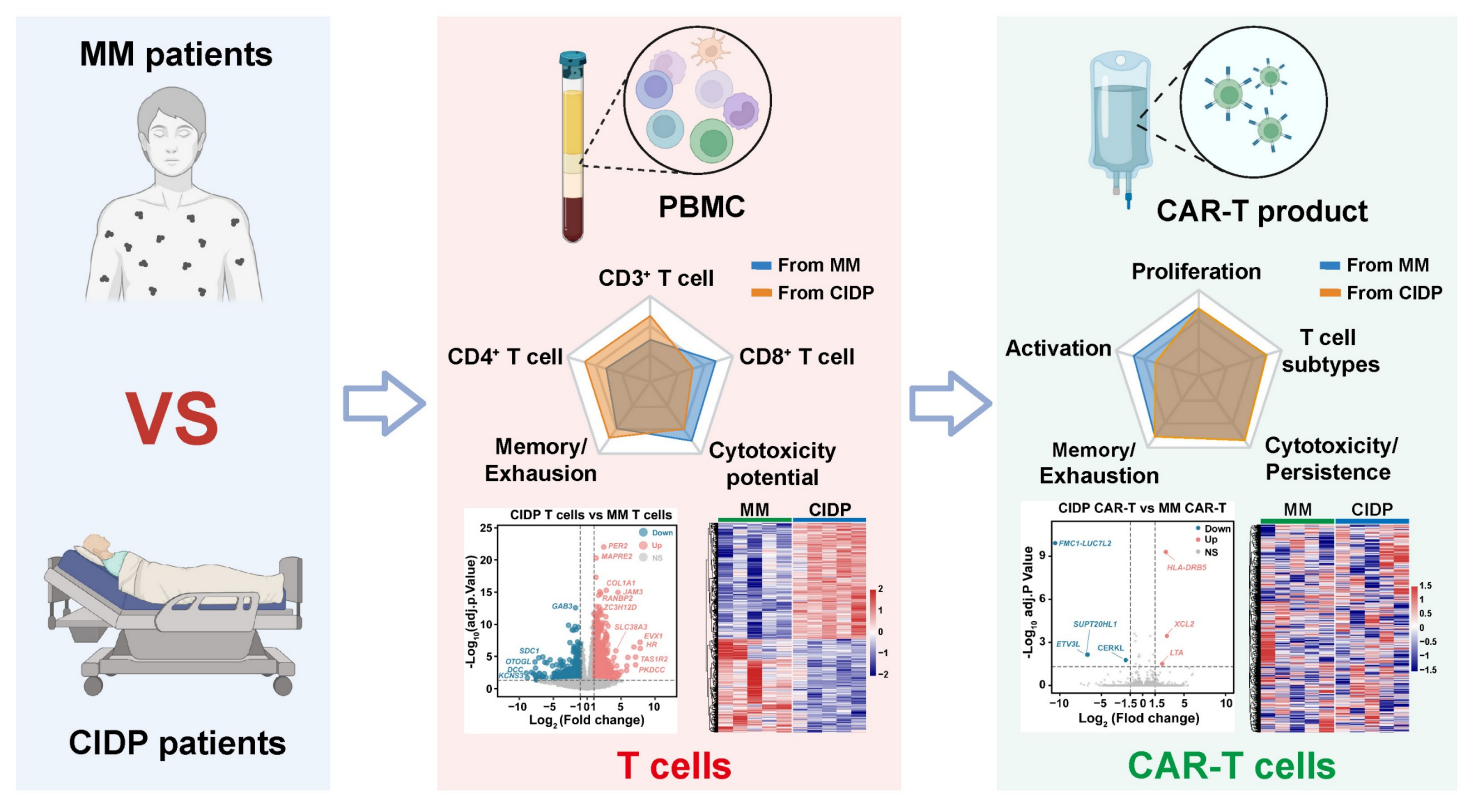
** Schematic of CAR-T manufacturing reducing T-cell heterogeneity across diseases.** The diagram depicts a comparative analysis of T cells derived from MM (malignancy) and CIDP (autoimmunity) cohorts, emphasizing disparities across phenotype, function, and the transcriptome. The CAR-T manufacturing process effectively reduces T-cell heterogeneity, resulting in products with convergent transcriptomic profiles and comparable *in vitro* cytotoxic potency against target cells.

Our study demonstrates that CAR-T cell manufacturing diminishes inherent T-cell heterogeneity across distinct disease contexts, specifically between immunosuppressed CIDP patients and cancer patients with MM. Despite obvious baseline differences in phenotypes and transcriptomic profiles, the manufacturing process yielded products with convergent differentiation states and preserved cytotoxic capacity **(Figure 7)**. This finding suggests that prolonged immunosuppression in autoimmune patients does not compromise T-cell fitness for adoptive cell therapy.

## Supplementary Material

Supplementary figures.

## Figures and Tables

**Table 1:** Information of MM and CIDP patients involved in this study

Disease	Patients	Gender	Age	Height (cm)	Weight (kg)	Disease stage	Treatment history
MM	MM-1	Male	65	170	52	ISS Stage III	VRD, autologous stem cell transplantation, BCMA CAR-T therapy, DARA, etc.
	MM-2	Female	57	155	60	ISS Stage III	Multiple chemotherapy → Disease progression
	MM-3	Female	63	162	52	ISS Stage II	PD, RD and PCDT*5, CDT*5
	MM-4	Male	69	170	60	ISS Stage II	VRD*4 courses--Autologous hematopoietic stem cell transplantation--VRD*2 courses--R maintenance*2 courses--Disease relapse --(Ixazomib + pomalidomide + dexamethasone) *1 course
	MM-5	Male	69	160	62	DS Stage III	Multiple chemotherapy → Disease progression
CIDP	CIDP-1	Male	33	175	74	None	High-dose Methylprednisolone pulse therapy (multiple rounds) →Methylprednisolone plus γ-globulin, > 1.5 years
	CIDP-2	Male	45	170	60	Acute exacerbation phase	Methylprednisolone → High-dose Methylprednisolone pulse therapy*2 → γ-globulin → Cyclophosphamide→ Rituximab, > 2 years
	CIDP-3	Male	43	175	76	CIDP	High-dose Methylprednisolone pulse therapy + γ-globulin → Prednisolone acetate, > 1 year
	CIDP-4	Male	73	180	80	Recurrent CIDP	High-dose Methylprednisolone pulse therapy + γ-globulin → Prednisolone acetate, > 1 year
	CIDP-5	Female	53	160	59	None	High-dose Methylprednisolone pulse therapy → Prednisolone acetate → γ-globulin, > 1.5 years

VRD, PD, RD, PCDT, CDT are the standard treatment regimens for MM. VRD: Velcade, Revlimid, Dexamethasone; PD: Pomalidomide, Dexamethasone; RD: Revlimid, Dexamethasone; PCDT: Pomalidomide, Cyclophosphamide, Dexamethasone, Thalidomide; CDT: Cyclophosphamide, Dexamethasone, Thalidomide; DARA: Daratumumab.

## Abbreviations

CAR-T: Chimeric antigen receptor T cell
ADs: Autoimmune diseases
HC: Healthy Control
MM: Multiple Myeloma
CIDP: Chronic inflammatory demyelinating polyneuropathy
SLE: Systemic lupus erythematosus
BCMA: Tumor necrosis factor receptor superfamily member 17
CD19: B-lymphocyte antigen CD19
BC19 CAR-T: BCMA and CD19 bispecific CAR-T
STR: Short tandem repeat
PMA: Phorbol 12-myristate 13-acetate
MOI: Multiplicity of infection
PBMC: Peripheral blood mononuclear cells
CIDP-CAR-T: CAR-T derived from CIDP patients
​​MM-CAR-T: CAR-T derived from MM patients
