# Supplementary material for: CAR-T manufacturing reduces heterogeneity between CIDP and multiple myeloma patient-derived T cells
Source: Theranostics. 2026 Jan 22;16(8):4245–59. doi: 10.7150/thno.125983 (PMC12905829; doi:10.7150/thno.125983)
Supplement: Supplementary file 1 — Supplementary figures. [file thnov16p4245s1.pdf]

Figure S1

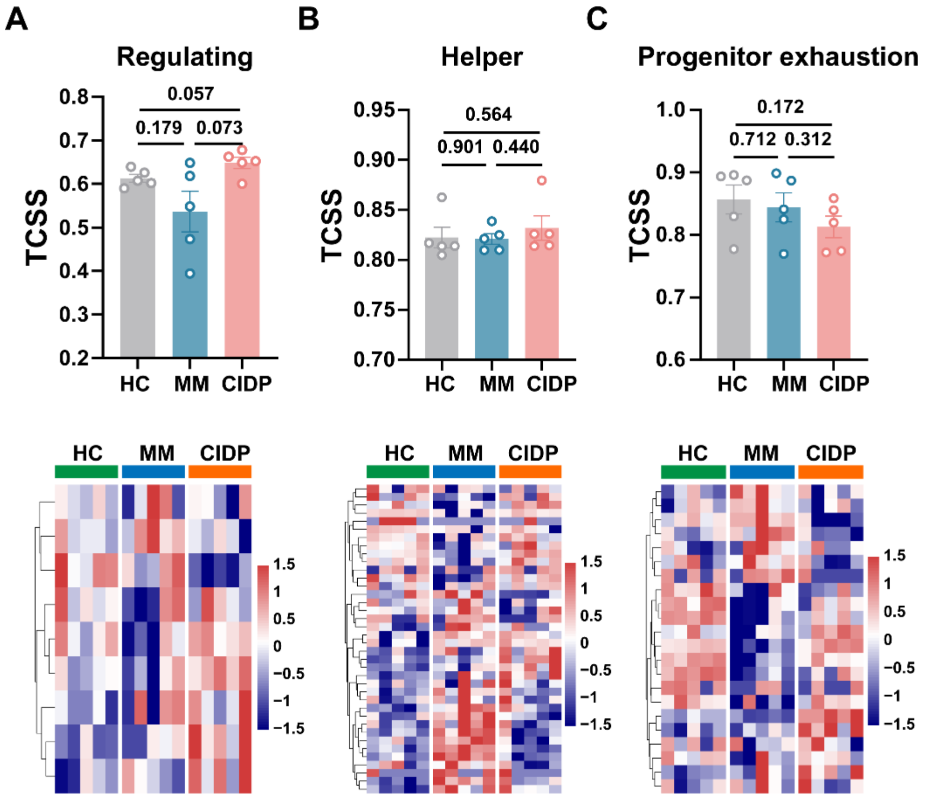

Figure S1. TCellSI and heatmaps of T cells derived from Healthy Control, MM and CIDP patients. TCellSI algorithm scores quantifying functional T cell states: Regulating, Helper, and Progenitor exhaustion. Heatmaps depicting expression levels of signature genes for selected T cell states: (A) Regulating, (B) Helper and (C) Progenitor exhaustion. Data are presented as mean ± SD. Statistical significance was set at $p < 0.05$.

Figure S2

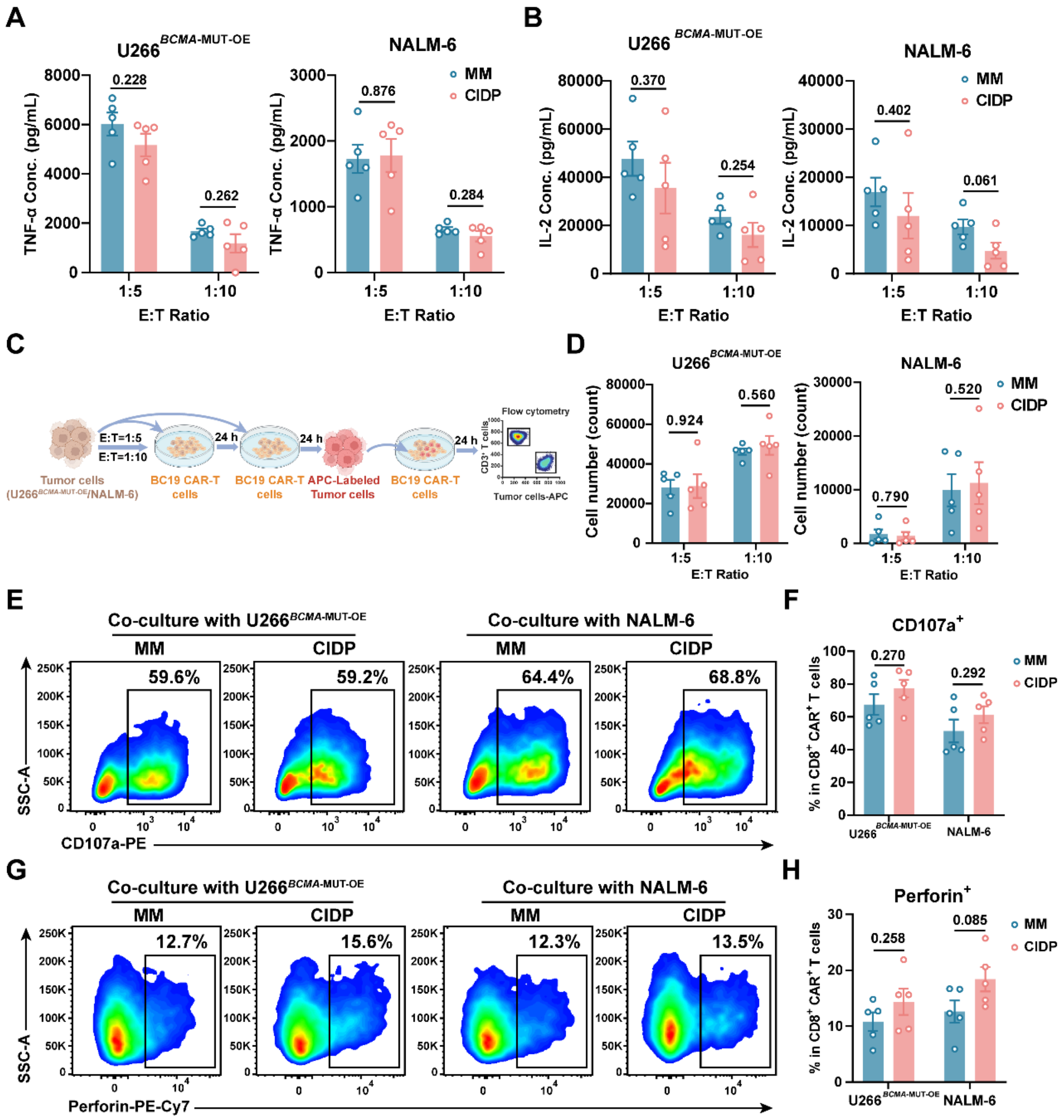

Figure S2. CIDP-CAR-T cells exhibit comparable cytotoxicity to MM-CAR-T cells upon antigen encounter. MM-CAR-T (n = 5) and CIDP-CAR-T (n = 5) cells were co-cultured with target cells expressing BCMA (U266$^{BCMA\text{-Mut-OE}}$) or CD19 (NALM-6). Secreted (A) TNF-α and (B) IL-2 levels in co-culture supernatants of U266$^{BCMA\text{-Mut-OE}}$ and NALM-6. Serial rechallenge assays assessed sustained cytotoxic function of MM-CAR-T (n = 5) and CIDP-CAR-T (n = 5) cells. (C) Schematic of sequential co-culture assay with fresh target cells (U266$^{BCMA\text{-mut-OE}}$ or NALM-6) added every 24 h for 3 rounds (R1-R3) at E : T ratios of 1:5 and 1:10. (D) Assessment of sustained

cytotoxicity in the third round. CAR-T cells were pre-challenged with unlabeled targets prior to the addition of fluorescently labeled targets in R3 to quantify specific lysis. Degranulation and perforin expression were measured after 4 h of co-culture. Representative flow plots and quantification of (E-F) $CD107a^+$ and (G-H) $Perforin^+$ cells within $CD8^+$ CAR^+ T cells. Data are presented as mean ± SD. Statistical significance was set at $p < 0.05$.

Figure S3

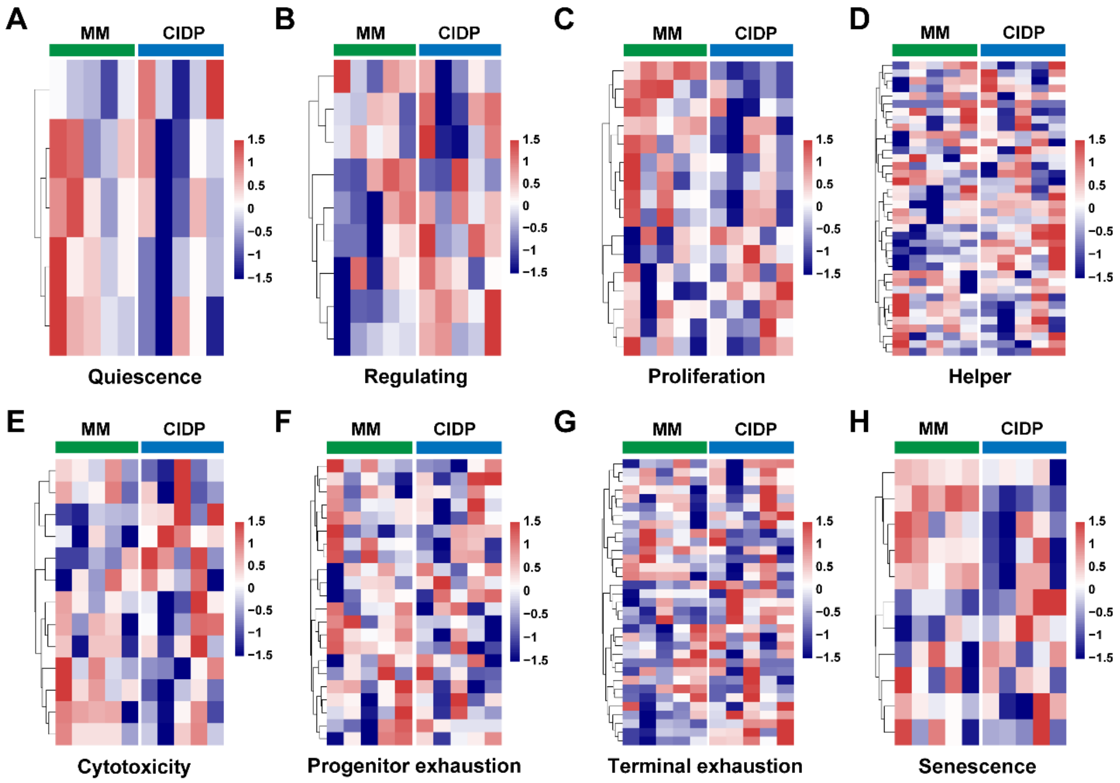

Figure S3. Heatmap of signature gene expression corresponding to CIDP-CAR-T and MM-CAR-T cell states.

Figure S4

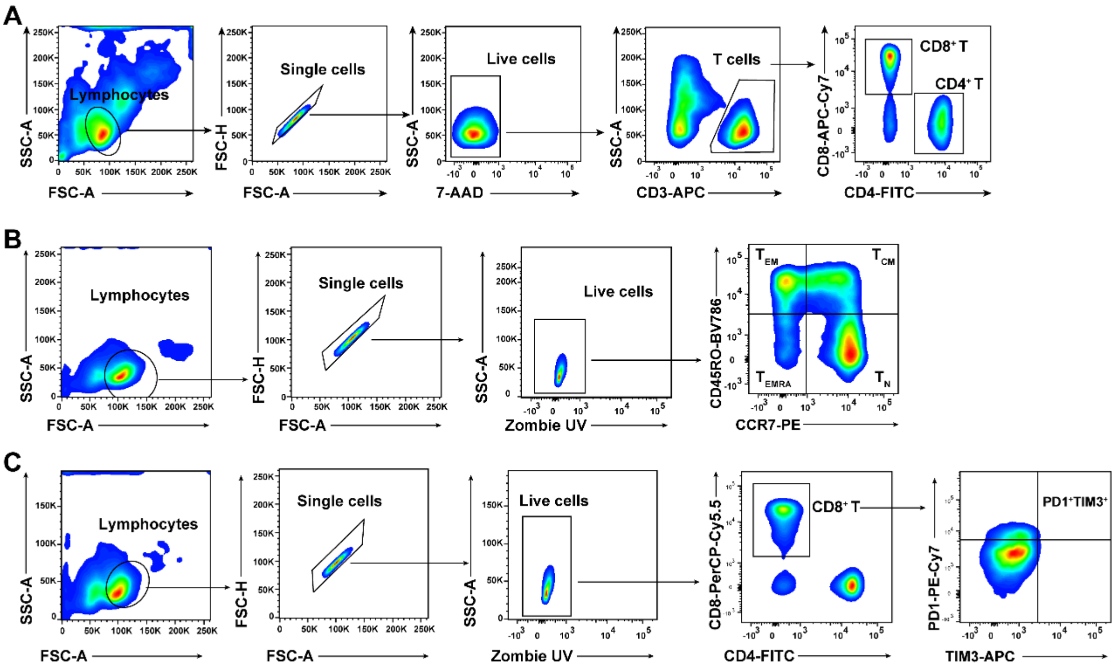

Figure S4. Gating strategy for T cell phenotype analysis. (A) Flow cytometry gating strategy for determining the proportions of CD3$^+$, CD4$^+$, and CD8$^+$ T cells in PBMCs from different sources. **(B)** Gating strategy for assessing memory phenotypes in T cells from different sources. **(C)** Gating strategy for evaluating exhaustion phenotypes in T cells from different sources.

Figure S5

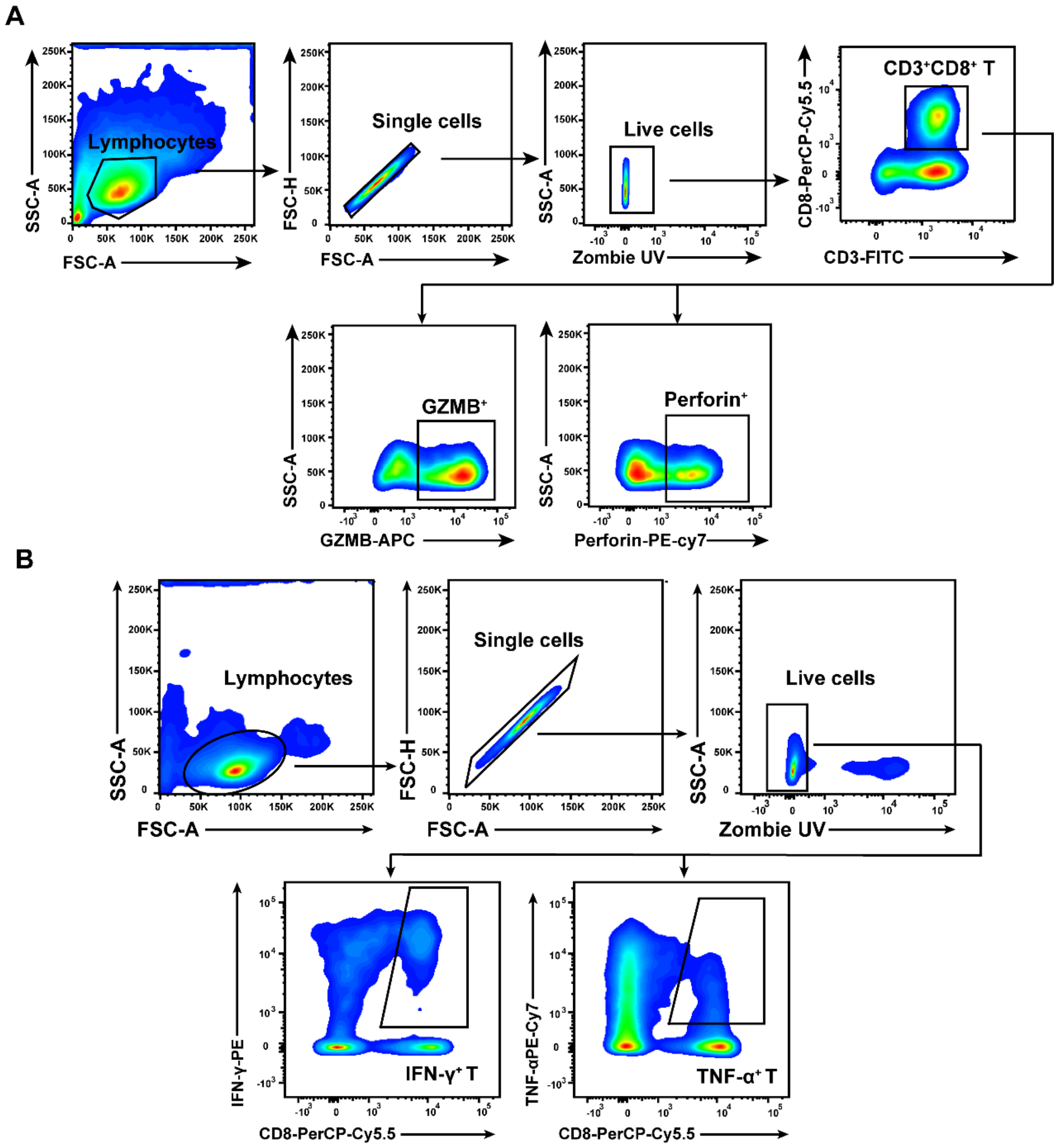

Figure S5. Gating strategy for T cell functional analysis. (A) Flow cytometry gating strategy for determining the proportions of Granzyme B^{+} and Perforin^{+} CD8^{+} T cells from different sources. (B) Flow cytometry gating strategy for determining the proportions of IFN-γ^{+} and TNF-α^{+} CD8^{+} T cells from different sources.

Figure S6

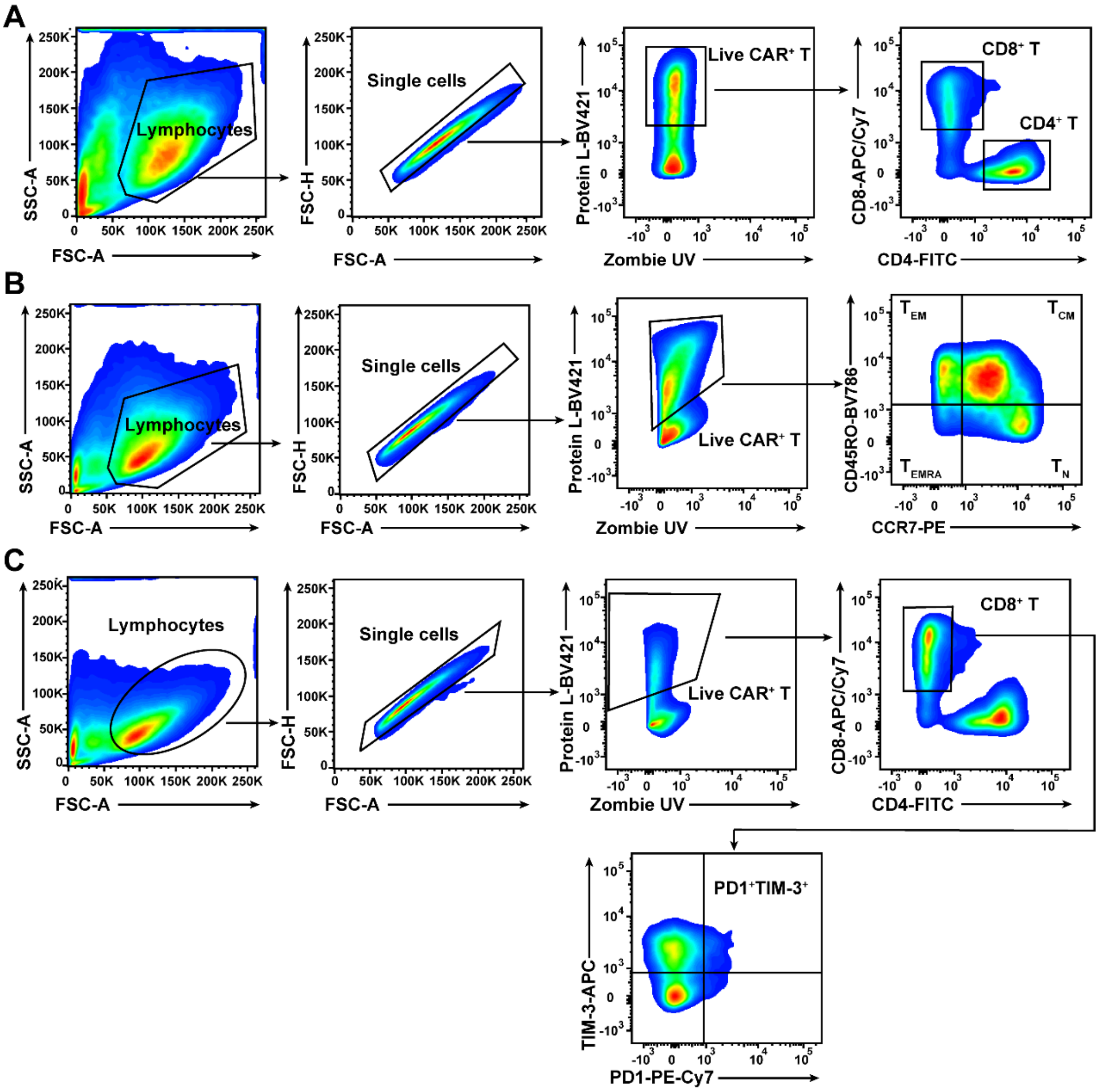

Figure S6. Gating strategy for CAR-T cell phenotype analysis. (A) Flow cytometry gating strategy for determining the proportions of $CD4^{+}$ and $CD8^{+}$ T cells in CAR-T from different sources. **(B)** Gating strategy for assessing memory phenotypes in CAR-T cells from different sources. **(C)** Gating strategy for evaluating exhaustion phenotypes in CAR-T cells from different sources.

Figure S7

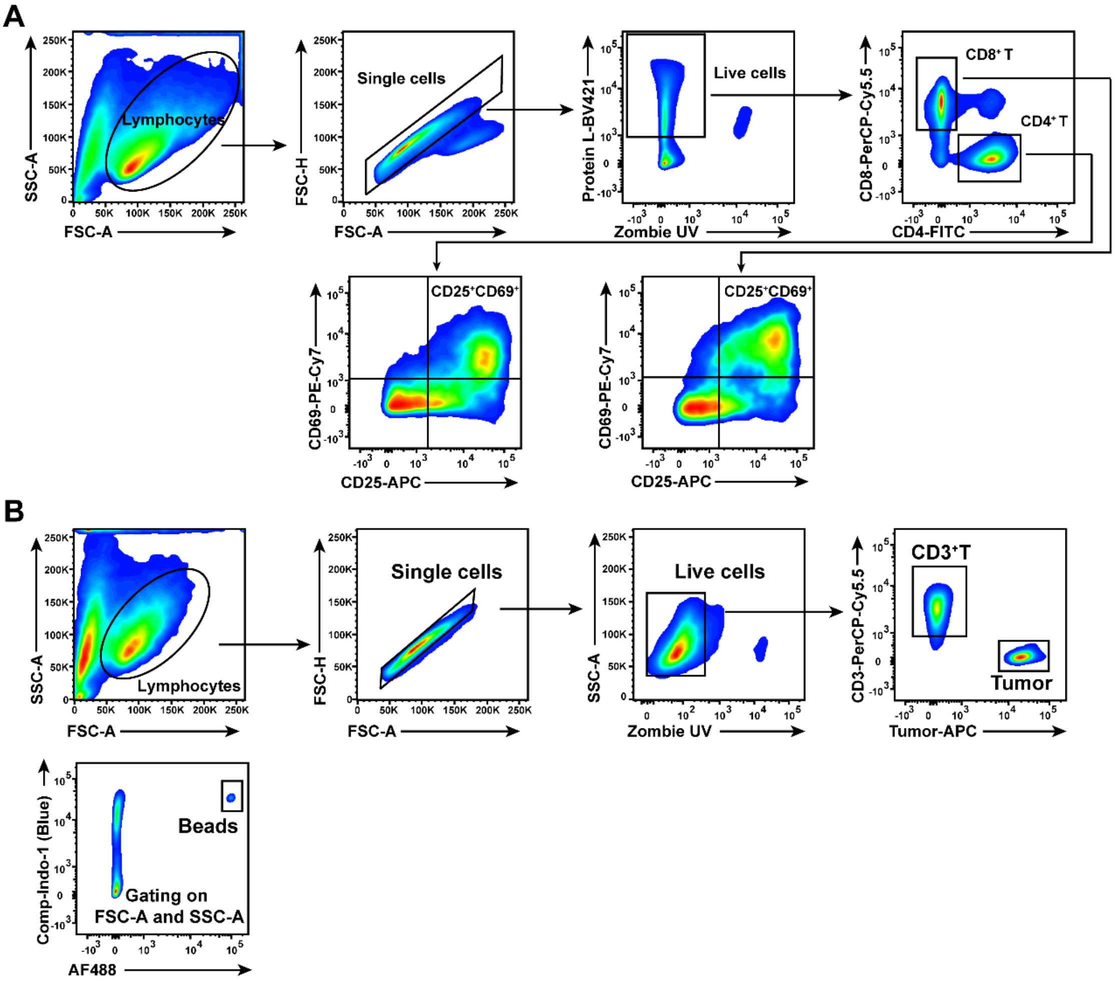

Figure S7. Gating strategy for CAR-T cell activation and cytotoxic function. (A) Flow cytometry gating strategy for assessing CAR-T cell activation following co-culture with antigen-positive tumor cells. **(B)** Flow cytometry gating strategy for evaluating CAR-T cell cytotoxicity against antigen-positive tumor cells following co-culture.

Figure S8

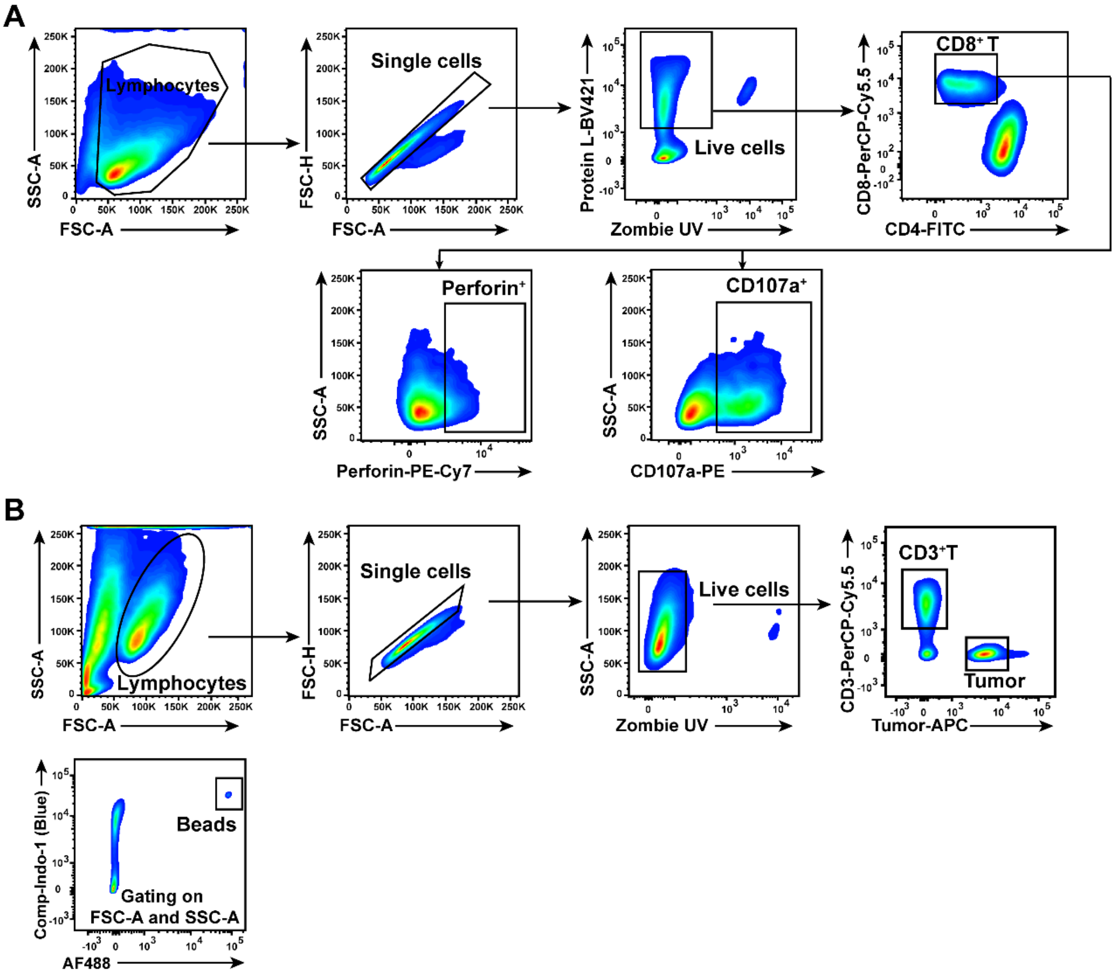

Figure S8. Gating strategy for CAR-T cell cytotoxic function. (A) Flow cytometry gating strategy for determining the proportions of CD107a^{+} and Perforin^{+} CD8^{+} CAR-T cells following co-culture with antigen-positive tumor cells. **(B)** Flow cytometry gating strategy for evaluating CAR-T cell cytotoxicity against target cells following multiple rounds of co-culture with antigen-positive tumor cells.
